# Supplementary figures and images for: A Focal Adhesion Filament Cross-correlation Kit for fast, automated segmentation and correlation of focal adhesions and actin stress fibers in cells
Source: PLoS One. 2021 Sep 10;16(9):e0250749. doi: 10.1371/journal.pone.0250749 (PMC8432882; doi:10.1371/journal.pone.0250749)

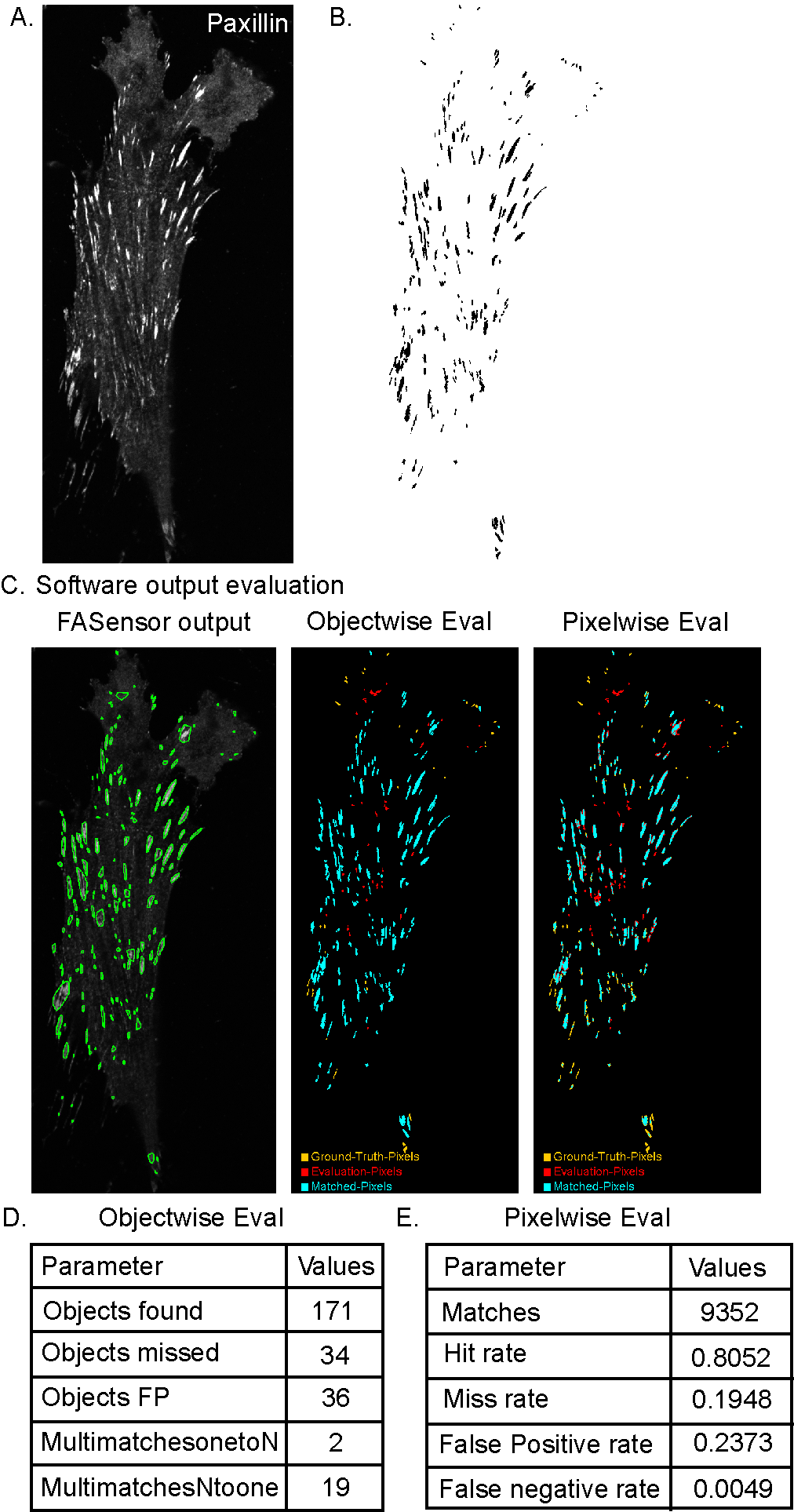

Supplement: S1 Fig — A) FASensor input of IF adhesion image showing the ventral plane of a MRC5 cell immunostained with paxillin. B) Binary mask of adhesion ROIs marked by user expert C) (Left-Right) Output adhesion objects detected by FASensor from input of A, Objectwise evaluation map of mask vs output, Pixelwise evaluation map of mask vs output. (in all evaluation images, found- blue, missed-yellow, false positive-red). D) Objectwise evaluation results tabulated. E) Pixelwise evaluation results tabulated. (PNG) [file pone.0250749.s001.png]

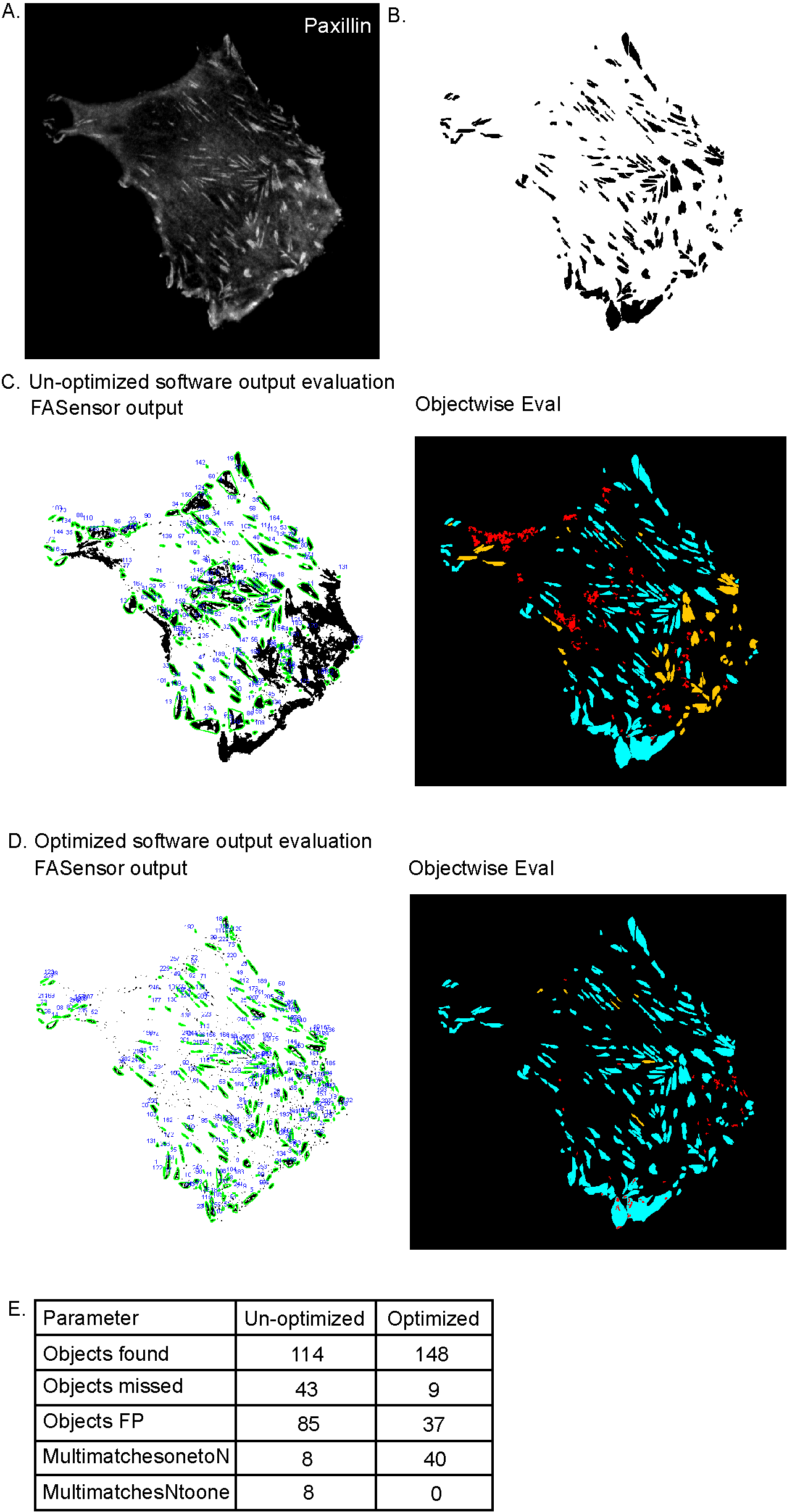

Supplement: S2 Fig — A) Grayscale image of adhesions, ventral plane of MRC5 cell immunostained for paxillin. B) Binary mask of adhesion ROIs manually marked by user expert from A through Fiji software. C) Un-optimized output vs user mask comparison (Left) FASensor output (Right) Objectwise evaluation map (found- blue, missed-yellow, false positive-red) D) Optimized output vs user mask comparison (Left) FASensor output (Right) Objectwise evaluation map (found- blue, missed-yellow, false positive-red) E) Table comparing results between un-optimized and optimized evaluations. (PNG) [file pone.0250749.s002.png]

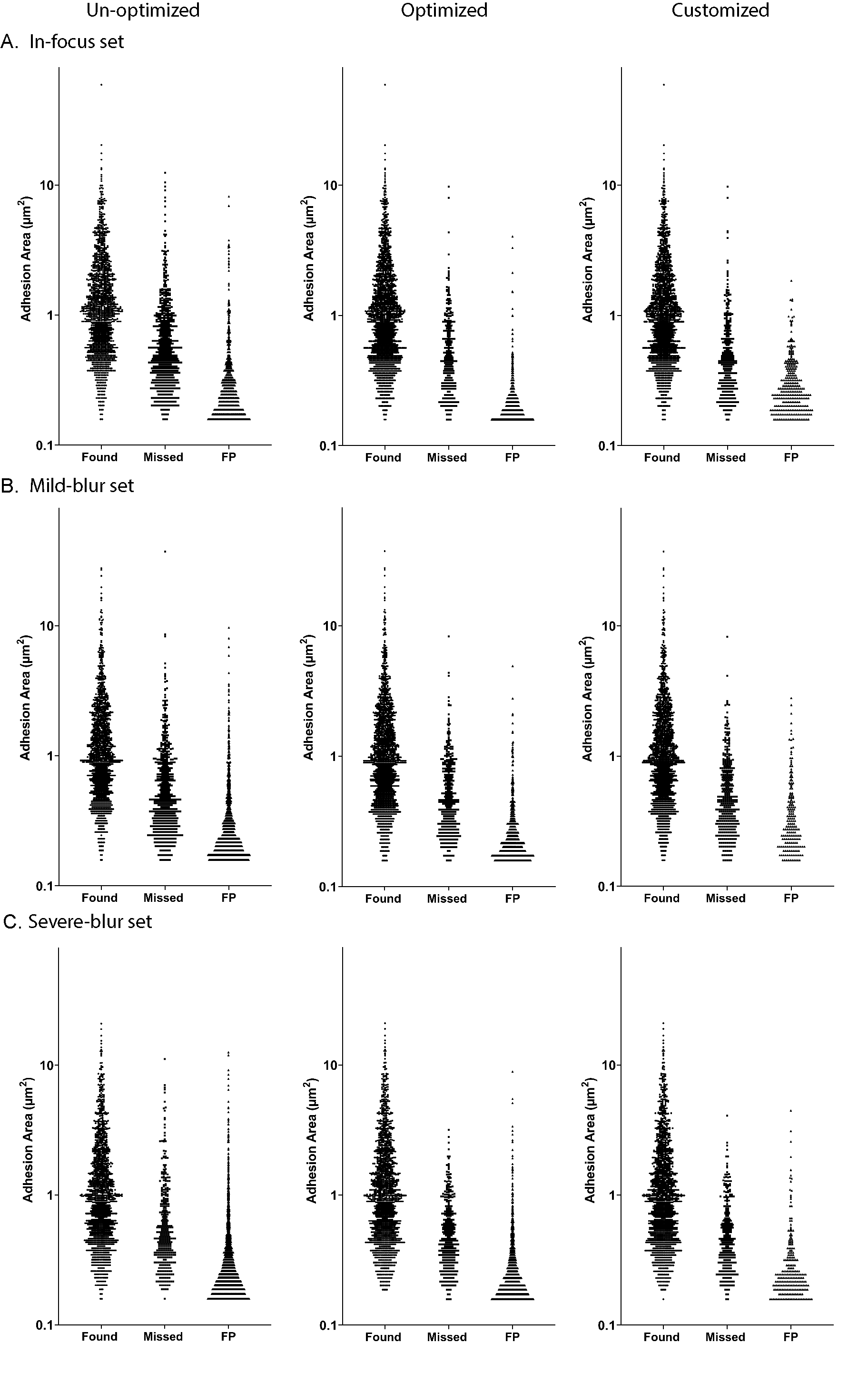

Supplement: S3 Fig — Graphs show pooled adhesion objects for found, missed and false positive (FP) categories in a set. Y axis is adhesion area in μm2 on a logarithmic scale. Left column shows un-optimized setting graphs, middle column shows optimized setting graphs and right column shows customized setting graphs. A) In focus set (Left) Found n = 1451 A¯=1.73μm2, Missed n = 660 A¯=0.86μm2 and FP n = 497 A¯=0.43μm2; (Middle) Found n = 1843 A¯=1.57μm2, Missed n = 268 A¯=0.71μm2 and FP n = 465 A¯=0.25μm2; (Right) Found n = 1802 A¯=1.59μm2, Missed n = 309 A¯=0.68μm2 and FP n = 296 A¯=0.33μm2; B) Mild-blur set (Left) Found n = 1522 A¯=1.64μm2, Missed n = 579 A¯=0.83μm2 and FP n = 791 A¯=0.42μm2; (Middle) Found n = 1740 A¯=1.57μm2, Missed n = 361 A¯=0.66μm2 and FP n = 560 A¯=0.31μm2; (Found n = 1700 A¯=1.60μm2, Missed n = 401 A¯=0.64μm2 and FP n = 188 A¯=0.43μm2; C) Severe-blur set (Left) Found n = 1504 A¯=1.61μm2, Missed n = 355 A¯=0.85μm2 and FP n = 1811 A¯=0.43μm2; (Middle) Found n = 1520 A¯=1.66μm2, Missed n = 339 A¯=0.61μm2 and FP n = 745 A¯=0.36μm2; (Right) Found n = 1577 A¯=1.62μm2, Missed n = 282 A¯=0.60μm2 and FP n = 231 A¯=0.36μm2. (PNG) [file pone.0250749.s003.png]

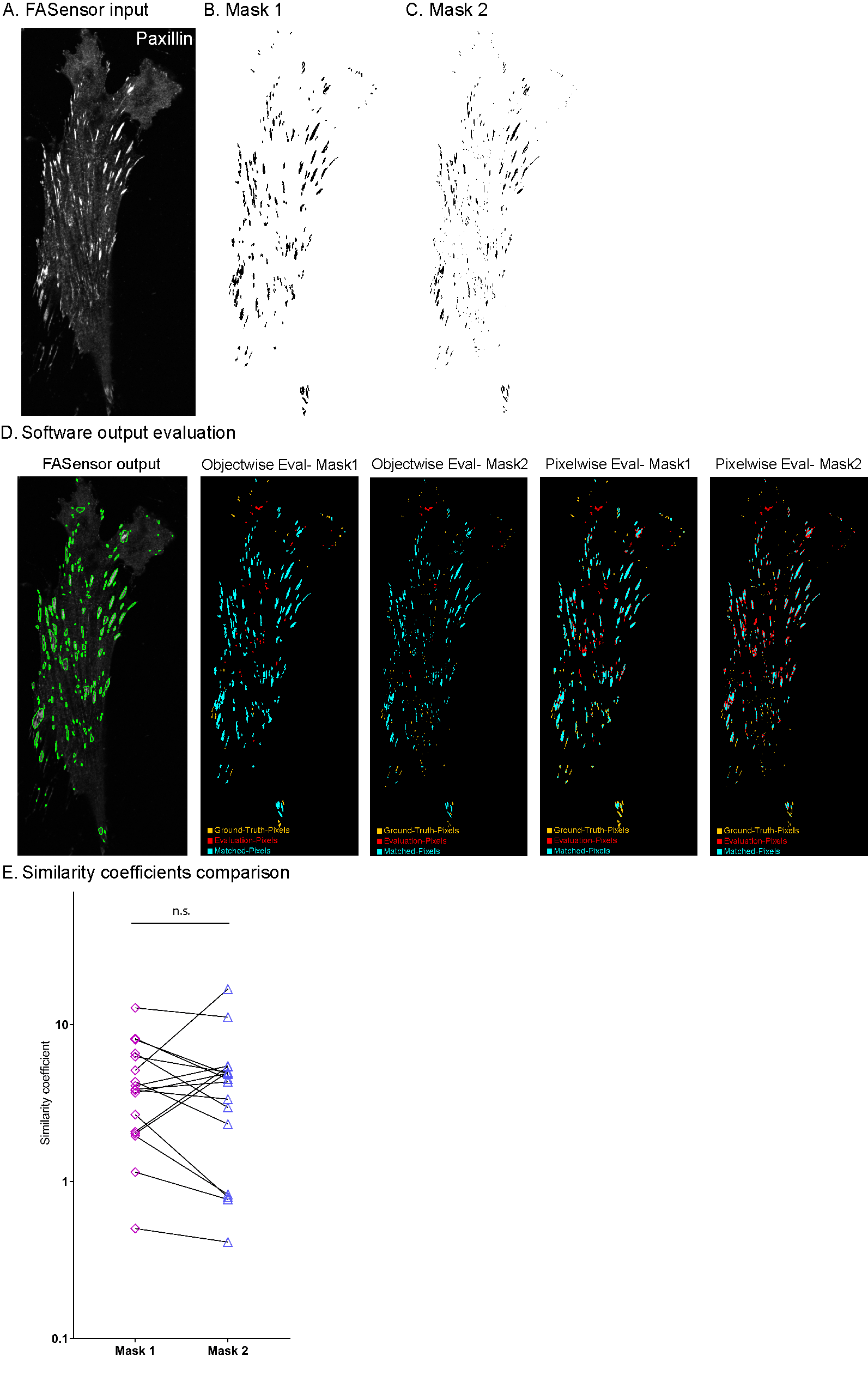

Supplement: S4 Fig — A) FASensor input of IF adhesion image showing the ventral plane of a MRC5 cell immunostained with paxillin. B) Binary mask of adhesion ROIs marked by user expert 1. C) Binary mask of adhesion ROIs marked by user expert 2. D) Evaluation output for pixel-wise and object-wise comparison of human expert annotations compared to FASensor output. E) Similarity coefficient of human expert annotated masks, human expert 1 (SC¯=4.53), human expert 2 (SC¯=4.63). (PNG) [file pone.0250749.s004.png]

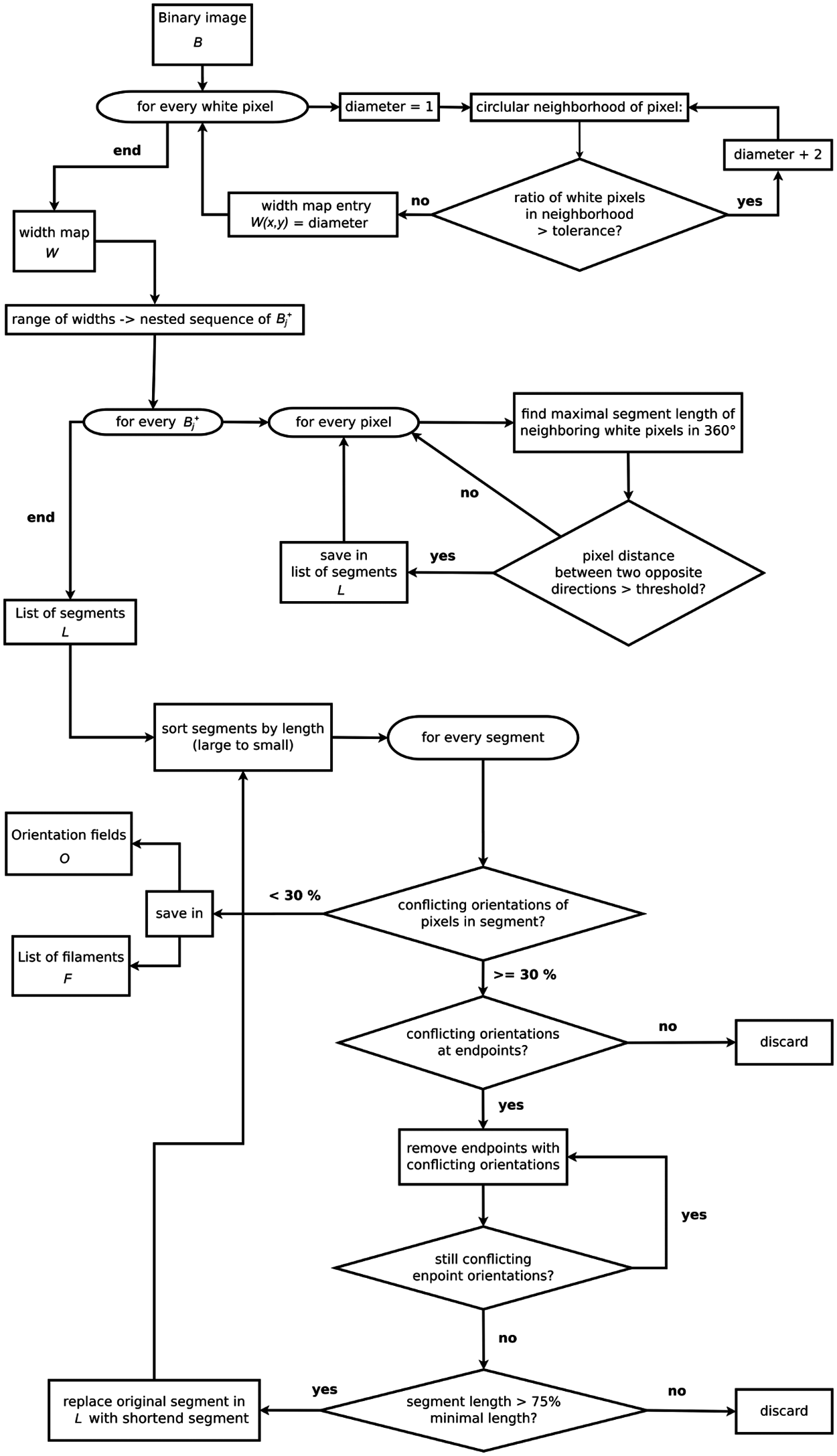

Supplement: S5 Fig — (PNG) [file pone.0250749.s005.png]
