# Supplementary material for: A Focal Adhesion Filament Cross-correlation Kit for fast, automated segmentation and correlation of focal adhesions and actin stress fibers in cells
Source: PLoS One. 2021 Sep 10;16(9):e0250749. doi: 10.1371/journal.pone.0250749 (PMC8432882; doi:10.1371/journal.pone.0250749)
Supplement: Attachment — Submitted filename: Response_to_reviewers.pdf [file pone.0250749.s007.pdf]

Response Letter to the Reviewers

Reviewer's Comments in black

Our answers in blue

Reviewer #1: This paper describes a new software package dedicated to quantifying the relationship between focal adhesions and stress fibers. Both of these structures are well studied and software to understanding how they interact from a quantitative perspective would be a nice addition. Overall, I like the concept for the paper and quite a bit of effort has been put in to test the capabilities of the software. I'm a bit concerned that the paper was submitted without any means to download or any instructions for how to use the software, but I think the authors can fix these problems.

We are happy about the positive feedback of the reviewer to our software package. About the concerns on missing instructions, we compiled installation notes, instructions, and a tutorial and will upload it to complement the software.

Major Issues:
I can't get your software to run on my computer. After downloading the jar file from zenodo, I ran:

~/Downloads$ java -jar GUIFocalAdhesionOnly.jar
Error: Could not find or load main class fa.view.MainWindow
Caused by: java.lang.NoClassDefFoundError: javafx/application/Application

This might be because I run Linux on my desktop. Since I didn't see any information in your paper about what operating systems you've tested, I wanted to bring this up as a potential issue. I could be making fundamental error as I don't often run java programs like this, but I couldn't find a README file either. Is this supposed to be a plugin for FIJI/ImageJ? If the other reviewers/editor didn't have any problem using the software then this can be disregarded.

We are very sorry that the referee could not run the software package. Unfortunately, the JavaFX package is not included in the main Java version anymore and has to be installed separately by the user and added to the path. Following the reviewers suggestions, we added detailed instructions in the installation notes how to install and add JavaFX as well as OpenJDK, if that is preferred over the Java distribution.

A section concerning software availability needs to be added to the materials and methods. This can be a short section, but it should detail where to download the software and on what operating systems it has been tested. Since the code is released under the GPL the source code should also be made available, preferable through github or a similar source code sharing website. The zenodo link provided should be integrated into this section.

We added the requested information to the main text including the link to the website (filament-sensor.de) where all tutorials, downloads, and ground databases can be found as well.

The description of the software interface on Lines 85-98 is confusing without a figure showing the software interface. I understand that you might not want to add a figure, but I find it strange to be reading a description of a software interface without actually having a figure to reference.

We are thankful for the hint that the description is somewhat confusing and edited paragraph for clarity. We added a figure showing an overview of the GUI (new Figure 3). Other views of the GUI and submenus not visible in this figure can be found in the tutorial (website and zenodo).

The description of optimizing the processing algorithm to match with a user input binary adhesion map from Lines 153-186 is really interesting, but I can't tell from the description what aspects of the algorithm were modified to optimize the result. Is this an automated process built into the software? In other words, can a user provide their ideal segmentation results and get back an algorithm parameter configuration to match up with the manual segmentation? This seems to be sort of covered in the materials and methods (Batch threshold determination...), but this section also doesn't detail what parameters are modified.

We are happy that the reviewer liked the optimization procedure and added more details about the optimization to the main text. We added the following section:

'FASensor output is derived in two ways: unoptimized (filter settings and thresholding that is default coded in the software and not necessarily appropriate for the cell) and optimized (appropriate filter settings and thresholding adjusted by the user). These settings can consist of pre-filters applied to the whole image, manually set thresholding algorithm parameters, applying or not applying the closing and fill holes options, setting boundary conditions for focal adhesion size, and finally separating focal adhesions via user input.'

My conclusion from the of the "FASensor output performance with varying imaging conditions and levels of optimization" section is that your software should not be used without a fair bit of effort being put in to customize the settings for every single image analyzed. I understand that settings need to be checked for a given set of images, but how bad is it to use the same settings for a set of images all gathered at the same time?

The similarity coefficient (SC) of software output to the user mask is higher when appropriate pre-processing filters and thresholding comprising the input routine is used as shown in Fig 5. While in the unoptimized (UN) case the software defaults are used, in the optimized (OP) analysis appropriate adjustments of input processing were introduced and furthermore in the customized case (CM) customization by the user in terms of deleting/editing adhesions. Specifically, for IF images that are in-focus, UN has a mean SC of 4.53 and is significantly ($p<0.05$) lower than OP at mean SC of 12.73. However, with images that are mildly blurred, the mean SC of UN (4.47) and OP (7.98) are not significantly different, same is true for images that have high blur (3.47 for UN vs 5.94 for OP). Thus, to answer the reviewer's question, one can achieve reliable analysis for a set of images using a single input setting across a dataset, through adding additional pre-processing steps that depend on the exact experiment (e.g. smoothing, adding Gaussian noise that would compare to the 'UN mild blur case').

That said, the advantage of the customizable settings is to adapt the software to the variety of different experimental conditions, instruments, and other parameters. Ideally, the user should customize for the specific settings (microscope type, fluorophore, noise, magnification, etc.) and then run batch processing for all images done in the same conditions.

Otherwise, I think this entire section comes down to the quality of the ground truth results. Since it seems like a single person was responsible for gathering these results, I'd like to see what the similarity coefficient is for independent manual segmentation results. Maybe you don't need to customize the settings at all because independent experts can't really decide what an FAs is in your sample images anyway.

To answer the question, we had another expert mark the in-focus dataset for adhesions and calculated the similarity coefficient with their masks for their unoptimized input routine in the software. The mean SC for this expert 2's set was 4.63, which is not significantly different from the expert 1's mean SC of 4.53 for the same input routine. This additional analysis is now added as a new Figure in the Supplemental Information.

The conclusion mentions analysis of dynamics (Line 389 and 397), but I don't see any evidence that the tool can handle dynamic or time-lapse analysis. I would expect that this would require tracking the stress fibers and FAs through time, as opposed to treating every image individually. These sections in the conclusion should be rewritten to indicate that the software could be extended to have this functionality.

We acknowledge the point raised by the reviewer and revised the concerning section in the following way: 'We are currently developing the functionality of the FAFCK such that it would be useful for analysis of time lapse movies, where many frames need to be analyzed consecutively with same settings to quantify the dynamics of stress fibers and adhesions in cells to understand their dynamic organization and how they influence the mechanical coupling of cells and the matrix.'

Currently we are also working on a tracking software for stress fibers that will be published soon and we are working on analyzing the focal adhesions from time-lapse recordings. However, this is beyond the scope of this paper and we clearly indicate that now in the manuscript.

Minor Issues:

Line 2: "cellular endoskeleton": I've never heard the cytoskeleton called an endoskeleton, but I don't suppose it's wrong.

We changed this to 'cytoskeleton'.

Line 16: "order parameter S": I'm not really sure what this means, is this a reference to a property you are going to calculate later in the paper?

The order parameter *S* is based on liquid crystal theory (as introduced by Zemel et al. Nat.

Phys 2010) for the quantitative analysis of the cytoskeleton. We added more details for clarity.

Line 130: “which is accurate with user’s expert perception of the IF image.” Maybe this should be a new sentence? Something like “After the reprocessing the user is able to confirm that the newly joined adhesion matches with expectations.”

We agree with the reviewer, split the sentence and slightly rephrased it.

Line 134: “between the user expert’s routine” Should this be the “user’s usual routine”?

Thank you for pointing this out. We changed the sentence.

Line 145: “with user mask” Should this be “with the user’s mask”?

Yes, indeed this was a typo – was corrected.

Line 298: “This serves as the ’artificial retina’” I really don’t know how the beginning of this sentence is related to the end. What is an artificial retina? Is the CMOS camera you used to gather the images an “artificial retina”? How does this help segment crossing filaments?

We apologize for using this ‘internal wording’ and edited the sentence for clarity.

Line 321: “Starting from the ends, for every point in a filament, every focal adhesion in the specified neighborhood is checked with the requirement that the focal adhesion main axis has to be longer than the filament length.” Does this sentence mean that the first filter tosses out any association between a FA with a shorter main axis as compared to the filament length? My intuition would be that nearly every filament will be longer than the associated FA, seeing as a large percentage of the filaments cover a substantial portion of the cell.

We thank the reviewer for pointing this out, this was an error in the manuscript. Indeed, we wanted to state: the focal adhesion main axis should be shorter than the filament length. We changed it accordingly.

Line 368: “help to answer many burning questions” I would strongly encourage the authors to consider not using the adjective burning here.

We changed the wording.

Line 374: “an quantification”, I think this should be “a quantification”
Line 378: “functionality allows to recognize” should be “functionality allows the recognition of”

Thanks for noting. We corrected these typos.

Line 378-381: “This software functionality allows to recognize different types of actin bundles in 2-dimensional images, such as maximum intensity projections of confocal stacks, which significantly quickens the quantification (in comparison with the necessity to detect structures in three-dimensional space, for example, dorsal stress fibers).”

This sentence is really difficult to follow, maybe something like this? “The software package can also be used to quantify maximum intensity projections from 3D image sets, making it possible to quickly quantify these difficult to measure structures.”

We changed the wording to clarify the sentence.

Reviewer #2: The authors reported an extended software module from the existing Focal Adhesion (FA) Sensor. This software provides an automated analysis of focal adhesion and Stress fiber. This software is multi-functional that fit the proposed purpose very well, also considered majority concerns of common images processing. The instruction is clear instruction and easy to follow. This software will benefit wide range of biological research that associated with Focal Adhesion and stress fiber. The manuscript is fairly completed in my opinion. Still, I have the following comments and would like to see the authors respond:

We are grateful and delighted that the reviewer appreciates our software and the future benefits for a wide range of biological questions. We are also very happy that the manuscript is considered ‘fairly completed’ and are happy to answer the remaining comments.

Major:
-Demonstrating biological relevance will provide a great substantiation of application of the software. Have the authors attempted to correlate the image analysis with migrating cell as the author hypothesized in discussion section? Or correlate the image analysis with experimentally measured mechanical properties of the MRC5 cell since FA and stress fiber play an important role for mechanosensing. The current imaging was only done in one culturing condition: MRC5 cells seeded on glass coverslips, which is a very rigid substrate.

We agree with the reviewer on biological relevance. However, this is beyond the scope of this paper, which is devoted to present the software as a tool. We do hope that it will be useful for many groups to use it for quantification of mechanosensing studies. That said we are currently working on a project where we employ this software to evaluate stress fiber organization and focal adhesions under variable conditions in a mechanistic study.

-Screen capture for the panels of the software for each corresponding operation will be helpful. Right now, the operation steps are all described solely by words, so the reader may not be able to fully appreciate the software.

We gratefully acknowledge this suggestion and added a screen shot of the GUI to the manuscript as new Fig. 3. Further screen shots can be found in the tutorial that is now included in the DOI. There, submenus that are not visible in the initial view, are described as well.

-Supplement Fig 3 seems to be an important evaluation of the software, I suggest to move it to main figure and combine with figure 4.

We agree with the reviewer about the importance and combined the figures. This is now the new Figure 5. Supp Fig 3 was removed.

Minor:
-Line 53 and 55: it is recommended to state what is “other methods” and “other tools” as the references cited are clear.

We added details and included citations.

-Fig 3C is presented before Fig 3A and B. I suggest to swap the order to match the presentation flow.

Thanks for noting. We changed the order.

-In general, the quality of the figures should be improved. From my end, most of the figures are pixelized, that include fluorescent images and the data figures. Especially for the legend in Fig 7C, that is un-readable.

We apologize for the poor quality. We have now enclosed higher resolution TIFF images of the figures.
