# Supplementary material for: A Focal Adhesion Filament Cross-correlation Kit for fast, automated segmentation and correlation of focal adhesions and actin stress fibers in cells
Source: PLoS One. 2021 Sep 10;16(9):e0250749. doi: 10.1371/journal.pone.0250749 (PMC8432882; doi:10.1371/journal.pone.0250749)
Supplement: Attachment — Submitted filename: Hauke et al.docx [file pone.0250749.s006.docx]

This paper describes a new software package dedicated to quantifying the relationship between focal adhesions and stress fibers. Both of these structures are well studied and software to understanding how they interact from a quantitative perspective would be a nice addition. Overall, I like the concept for the paper and quite a bit of effort has been put in to test the capabilities of the software. I’m a bit concerned that the paper was submitted without any means to download or any instructions for how to use the software, but I think the authors can fix these problems.

Major Issues:

I can’t get your software to run on my computer. After downloading the jar file from zenodo, I ran:

~/Downloads$ java -jar GUIFocalAdhesionOnly.jar

Error: Could not find or load main class fa.view.MainWindow

Caused by: java.lang.NoClassDefFoundError: javafx/application/Application

This might be because I run Linux on my desktop. Since I didn’t see any information in your paper about what operating systems you’ve tested, I wanted to bring this up as a potential issue. I could be making fundamental error as I don’t often run java programs like this, but I couldn’t find a README file either. Is this supposed to be a plugin for FIJI/ImageJ? If the other reviewers/editor didn’t have any problem using the software then this can be disregarded.

A section concerning software availability needs to be added to the materials and methods. This can be a short section, but it should detail where to download the software and on what operating systems it has been tested. Since the code is released under the GPL the source code should also be made available, preferable through github or a similar source code sharing website. The zenodo link provided should be integrated into this section.

The description of the software interface on Lines 85-98 is confusing without a figure showing the software interface. I understand that you might not want to add a figure, but I find it strange to be reading a description of a software interface without actually having a figure to reference.

The description of optimizing the processing algorithm to match with a user input binary adhesion map from Lines 153-186 is really interesting, but I can’t tell from the description what aspects of the algorithm were modified to optimize the result. Is this an automated process built into the software? In other words, can a user provide their ideal segmentation results and get back an algorithm parameter configuration to match up with the manual segmentation? This seems to be sort of covered in the materials and methods (Batch threshold determination... ), but this section also doesn’t detail what parameters are modified.

My conclusion from the of the “FASensor output performance with varying imaging conditions

and levels of optimization” section is that your software should not be used without a fair bit of effort being put in to customize the settings for every single image analyzed. I understand that settings need to be checked for a given set of images, but how bad is it to use the same settings for a set of images all gathered at the same time? Otherwise, I think this entire section comes down to the quality of the ground truth results. Since it seems like a single person was responsible for gathering these results, I’d like to see what the similarity coefficient is for independent manual segmentation results. Maybe you don’t need to customize the settings at all because independent experts can’t really decide what an FAs is in your sample images anyway.

The conclusion mentions analysis of dynamics (Line 389 and 397), but I don’t see any evidence that the tool can handle dynamic or time-lapse analysis. I would expect that this would require tracking the stress fibers and FAs through time, as opposed to treating every image individually. These sections in the conclusion should be rewritten to indicate that the software could be extended to have this functionality.

Minor Issues:

Line 2: “cellular endoskeleton”: I’ve never heard the cytoskeleton called an endoskeleton, but I don’t suppose it’s wrong.

Line 16: “order parameter S”: I’m not really sure what this means, is this a reference to a property you are going to calculate later in the paper?

Line 130: “which is accurate with user’s expert perception of the IF image.” Maybe this should be a new sentence? Something like “After the reprocessing the user is able to confirm that the newly joined adhesion matches with expectations.”

Line 134: “between the user expert’s routine” Should this be the “user’s usual routine”?

Line 145: “with user mask” Should this be “with the user’s mask”?

Line 298: “This serves as the ’artificial retina’” I really don’t know how the beginning of this sentence is related to the end. What is an artificial retina? Is the CMOS camera you used to gather the images an “artificial retina”? How does this help segment crossing filaments?

Line 321: “Starting from the ends, for every point in a filament, every focal adhesion in the

specified neighborhood is checked with the requirement that the focal adhesion main

axis has to be longer than the filament length.” Does this sentence mean that the first filter tosses out any association between a FA with a shorter main axis as compared to the filament length? My intuition would be that nearly every filament will be longer than the associated FA, seeing as a large percentage of the filaments cover a substantial portion of the cell.

Line 368: “help to answer many burning questions” I would strongly encourage the authors to consider not using the adjective burning here.

Line 374: “an quantification”, I think this should be “a quantification”

Line 378: “functionality allows to recognize” should be “functionality allows the recognition of”

Line 378-381: “This software functionality allows to recognize different types of actin bundles in

2-dimensional images, such as maximum intensity projections of confocal stacks, which

significantly quickens the quantification (in comparison with the necessity to detect

structures in three-dimensional space, for example, dorsal stress fibers ).”

This sentence is really difficult to follow, maybe something like this? “The software package can also be used to quantify maximum intensity projections from 3D image sets, making it possible to quickly quantify these difficult to measure structures.”
